# Overexpression of pyruvate dehydrogenase kinase 1 in retinoblastoma: A potential therapeutic opportunity for targeting vitreous seeds and hypoxic regions

**DOI:** 10.1371/journal.pone.0177744

**Published:** 2017-05-15

**Authors:** Swatishree Sradhanjali, Devjyoti Tripathy, Suryasnata Rath, Ruchi Mittal, Mamatha M. Reddy

**Affiliations:** 1 The Operation Eyesight Universal Institute for Eye Cancer, L V Prasad Eye Institute, Bhubaneswar, India; 2 School of Biotechnology, KIIT University, Bhubaneswar, India; 3 Ophthalmic Plastics, Orbit and Ocular Oncology Services, L V Prasad Eye Institute, Bhubaneswar, India; 4 Dalmia Ophthalmic Pathology Services, LV Prasad Eye Institute, Bhubaneswar, India; University of South Alabama Mitchell Cancer Institute, UNITED STATES

## Abstract

Pyruvate dehydrogenase kinase 1 (PDK1), a key enzyme implicated in metabolic reprogramming of tumors, is induced in several tumors including glioblastoma, breast cancer and melanoma. However, the role played by PDK1 is not studied in retinoblastoma (RB). In this study, we have evaluated the expression of PDK1 in RB clinical samples, and studied its inhibition as a strategy to decrease cell growth and migration. We show that PDK1 is specifically overexpressed in RB patient samples especially in vitreous seeds and hypoxic regions and cell lines compared to control retina using immunohistochemistry and real-time PCR. Our results further demonstrate that inhibition of PDK1 using small molecule inhibitors dichloroacetic acid (DCA) and dichloroacetophenone (DAP) resulted in reduced cell growth and increased apoptosis. We also confirm that combination treatment of DCA with chemotherapeutic agent carboplatin further enhanced the therapeutic efficacy compared to single drug treatment. In addition, we observed changes in glucose uptake, lactate and reactive oxygen species (ROS) levels as well as decreased cell migration in response to PDK1 inhibition. Additionally, we show that DCA treatment led to inhibition of PI3K/Akt pathway and reduction in PDK1 protein levels. Overall, our data suggest that targeting PDK1 could be a novel therapeutic strategy for RB.

## Introduction

Retinoblastoma (RB) is the most common intraocular malignancy in children below the age of five years. The incidence of RB ranges from 1/15,000 to 1/18,000 live births and it can either be bilateral or unilateral. Inactivating mutations in RB1 gene are an initiating event in most cases of RB. Yet, additional events are required, further to RB1 mutations, for manifestation of RB [1,2]. Molecular analysis of RB tumors revealed that epigenetic deregulation plays a major role in the tumorigenesis [3]. However, recently, presence of MYCN amplification has been reported in a subset of RB patients with no apparent alterations in RB1 [4]. Though, recent developments in the field led to increased cure rates, advanced disease presentation is still a problem in developing nations [5]. Despite chemotherapy is available for RB, not all children respond and treatment especially, is a challenge in tumors with hypoxic regions and vitreous seeds [6].

Presence of hypoxia has been demonstrated in mouse RB tumors [7]. Similarly, hypoxic regions were also observed in human RB tumors and expression of Hypoxia Inducible Factor 1 Alpha (HIF1α) was found in 83% of human RB specimens [8]. Consequently, it is necessary to focus on biochemical pathways that get activated in vitreous seeds and hypoxic conditions to develop more targeted approaches. Tumor cell metabolism is one of the several processes regulated by hypoxia. Cancer cells alter their metabolism to meet the increased demand for biosynthetic substrates required for uncontrolled cell proliferation.

Several rate-limiting metabolic enzymes have been recognized to play a critical role in tumor cell transformation. PDK1 is one such key enzyme that has been showed to play a central role in metabolic reprogramming in various tumors [9]. PDK1 catalyzes the phosphorylation of pyruvate dehydrogenase (PDH) and inactivates it. PDH converts pyruvate into acetyl CoA that is further metabolized in mitochondria via Krebs' cycle. The role of PDK1 has not been studied so far in RB. In the current study, we present data to show that PDK1 was overexpressed in human RB tumor specimens, and targeting PDK1 resulted in decreased cell growth and migration in retinoblastoma-derived cell lines.

## Materials and methods

### Reagents

Dichloroacetic acid (DCA), 2',7'-dichlorodihydrofluorescein diacetate (DCF-DA), Dichloroacetophenone (DAP) and cobalt chloride were purchased from Sigma-Aldrich (Bengaluru, Karnataka, India) and used at various concentrations in the study. Cobalt chloride solution was made freshly before use. Glucose analogue 2-(N-(7-Nitrobenz-2-oxa-1,3-diazol-4-yl)Amino)-2-Deoxyglucose (2-NBD glucose) was obtained from Cayman chemical, Ann Arbor, MI, USA. Trizol from Life Technologies (Carlsbad, CA, USA) was used to isolate RNA and reverse transcription kit was from Thermo Scientific (Waltham, MA, USA). Rabbit polyclonal PDK1 antibody, rabbit monoclonal HIF1α, rabbit monoclonal phospho-AKT (Thr308) and rabbit monoclonal Akt antibodies were purchased from Cell Signaling (Danvers, MA, USA) and β-actin antibody was from Sigma-Aldrich. Apoptosis kit was procured from Roche (Basel, Switzerland). Poly-L- lysine (0.1%, Sigma Aldrich) was made to a final concentration of 0.01% to coat the 6-well cell culture dishes.

### Immunohistochemistry (IHC)

The study was approved by the ethics committee of LV Prasad Eye Institute, Bhubaneswar and conducted according to the declaration of Helsinki. Tissue sections from enucleated eyes were used to study the expression of PDK1. Human RB tissues were fixed in formalin and embedded in paraffin wax. The formalin-fixed paraffin-embedded (FFPE) tissues were sectioned on microtome and placed on coated microscopic slides. The sections were deparaffinized and further processed for Hematoxylin and Eosin (H&E) staining and IHC. Anti-PDK1 antibody was used at a dilution of 1:50 for the detection of PDK1 expression.

### Determination of mRNA expression

RNA extracted using Trizol reagent was quantified by biospectrophotometer (Eppendorf, Hamburg, Germany). RNA was stored at -80°C till further analysis. cDNA was synthesized using reverse transcription reagents as per the manufacturer’s instructions. Real time PCR analyses were performed using power SYBR® Green PCR master mix in triplicates. β2-microglobulin was employed as an endogenous control. The following gene specific primers were used: β2-microglobulin, forward: 5′-GGTTGGCCAATCTACTCCCAGG-3′ and reverse: 5′-CAACTTCATCCACGTTCACC-3′ (Eurofins, Kolkata, India); PDK1, forward: 5′-CAACAGAGGTGTTTACCCCC-3′ and reverse: 5′-ATTTTCCTCAAAGGAACGCC-3′.

### Cell culture

Human retinoblastoma cell lines Y79 and Weri-Rb1 and human retinal pigment epithelial cells ARPE-19 were purchased from ATCC. Y79 and Weri-Rb1 cells were maintained in RPMI-1640 containing 2 mM L-Glutamine, 10% Fetal Bovine Serum (FBS) and 1% penicillin-streptomycin-amphotericin B mixture at 37°C and 5% CO_2_. To mimic hypoxic environment, cells were maintained under hypoxic condition (with 0.5% O_2_ level) or treated with cobalt chloride (100, 200 and 300 μM). ARPE-19 cells were maintained in DMEM-F12 medium containing 10% FBS. RB tumor tissues (LRB1, LRB2) were collected from enucleated eyes after obtaining written informed consent from parents of the patients in accordance with the ethical protocols of our institute and declaration of Helsinki. The RB patient samples were maintained in same culture conditions as described earlier for cell lines. The LRB1 and LRB2 primary cell cultures were derived from tumors that were largely hypoxic and contained vitreous seeds. The tumor cells were verified to be RB negative. In addition, these primary cultures show comparable morphology and growth characteristics similar to commercially available RB cell lines Y79 and Weri-Rb1.

### Inhibition of PDK1 with DCA and DAP

Cells were seeded at a low density (0.1x10^6^ cells/ml) in T25 tissue culture flasks and treated with various concentrations of DCA (1, 3 and10 mM) or DAP (1, 3, 10, 30 and 60 μM). The cell growth was studied by MTS assay (Promega, Madison, WI, USA). Each experiment was repeated at least thrice.

### Immunoblotting

Immunoblotting was performed as described previously [10]. The antibodies used in the study include rabbit polyclonal anti-PDK1, rabbit monoclonal anti-phospho-Akt (Thr308), rabbit monoclonal anti-Akt, rabbit monoclonal anti-HIF1α and mouse monoclonal anti-β-actin. Standard chemiluminescence was employed for the detection of protein of interest.

### Apoptosis

Cells were treated with DCA (10 mM) for 48 hours and apoptosis was evaluated by using Annexin V Fluos kit (Roche) as per manufacturer’s instructions.

### Glucose uptake assay

The glucose uptake in response to PDK1 inhibition was determined by flow cytometry using a fluorescent glucose analogue, 2-NBD-glucose as described before [11]. In brief, cells were treated with 3 mM DCA for 48 hours and measured glucose uptake. Equal number of live cells (1x10^6^) were incubated with 2-NBD-glucose (50 μM) in phosphate buffered saline (PBS) for 30 min at 37°C, washed twice in cold PBS and analyzed by flow cytometry. Relative glucose uptake after DCA treatment was calculated based on differences in fluorescence compared to control cells. The results were normalized to cell viability.

### Measurement of reactive oxygen species (ROS)

The relative levels of ROS were measured using redox sensitive fluorochrome, DCF-DA (2',7'-dichlorodihydrofluorescein diacetate) as described previously [12]. Approximately, 1x10^6^ cells were washed twice with sterile PBS and incubated with 20°M DCF-DA for 25 min at 37°C. Following incubation, cells were washed twice in cold PBS and analyzed for ROS using flow cytometer (BD FACS Calibur, San Jose, CA, USA). Differences in ROS levels were determined and compared to untreated controls after normalizing the cell viability. In additional experiments, Y79 cells were treated with antioxidant vitamin C (500 μM) and/or DCA and ROS levels and cell growth were measured.

### Migration assay

The migration assay was performed as described previously [13]. Cells (1x10^6^) were seeded in poly-L-lysine (0.01%) coated 6-well tissue culture dishes and maintained in RPMI-1640 medium supplemented with 1% FBS until the cells reached 75–100% confluence. Fine scratches were made using a 200 μL sterile pipette tip. Cells were washed twice with PBS and cultured in fresh medium containing 10% FBS in the presence of desired DCA concentrations. Cells were incubated at 37°C and 5% CO_2_ for 18 hours. The scratch created was photographed at various time points.

### Drug combination assays

Synergy analysis was performed using a method proposed by Chou and Talalay [14]. Briefly; cells were treated with either DCA or carboplatin alone and also with a combination of DCA and carboplatin. For drug combination studies, cells were sensitized with DCA for 24 hours and then treated with carboplatin for 48 hours. Initially, different concentrations of drugs were chosen to study the growth inhibition using MTS assay and IC_50_ value of individual drug was determined. Later, drug concentrations below IC_50_ were chosen to study the combination effect of drugs. Percentage of growth in treated samples was calculated compared to control and fraction affected (FA) was calculated by using the formula: FA = 1-(% growth/100). FA values for each concentration of single drug (DCA or carboplatin) and the combinations were entered into the compusyn program. Combination index (CI) was determined for each drug combination. CI value less than 1 is considered as synergistic, equal to 1 as additive and more than 1 as antagonistic.

### Statistical analysis

Percentage of change in treatment groups relative to the average of the control group was calculated. The quantitative data represent mean of at least three independent experiments and error bars indicate standard deviation. The statistical differences between treatment and control groups were analyzed using the ANOVA or Student’s t-test. The p values less than 0.05 were considered significant.

## Results

### PDK1 is strongly expressed in vitreous seeds and hypoxic regions of human RB

**Fig 1:**
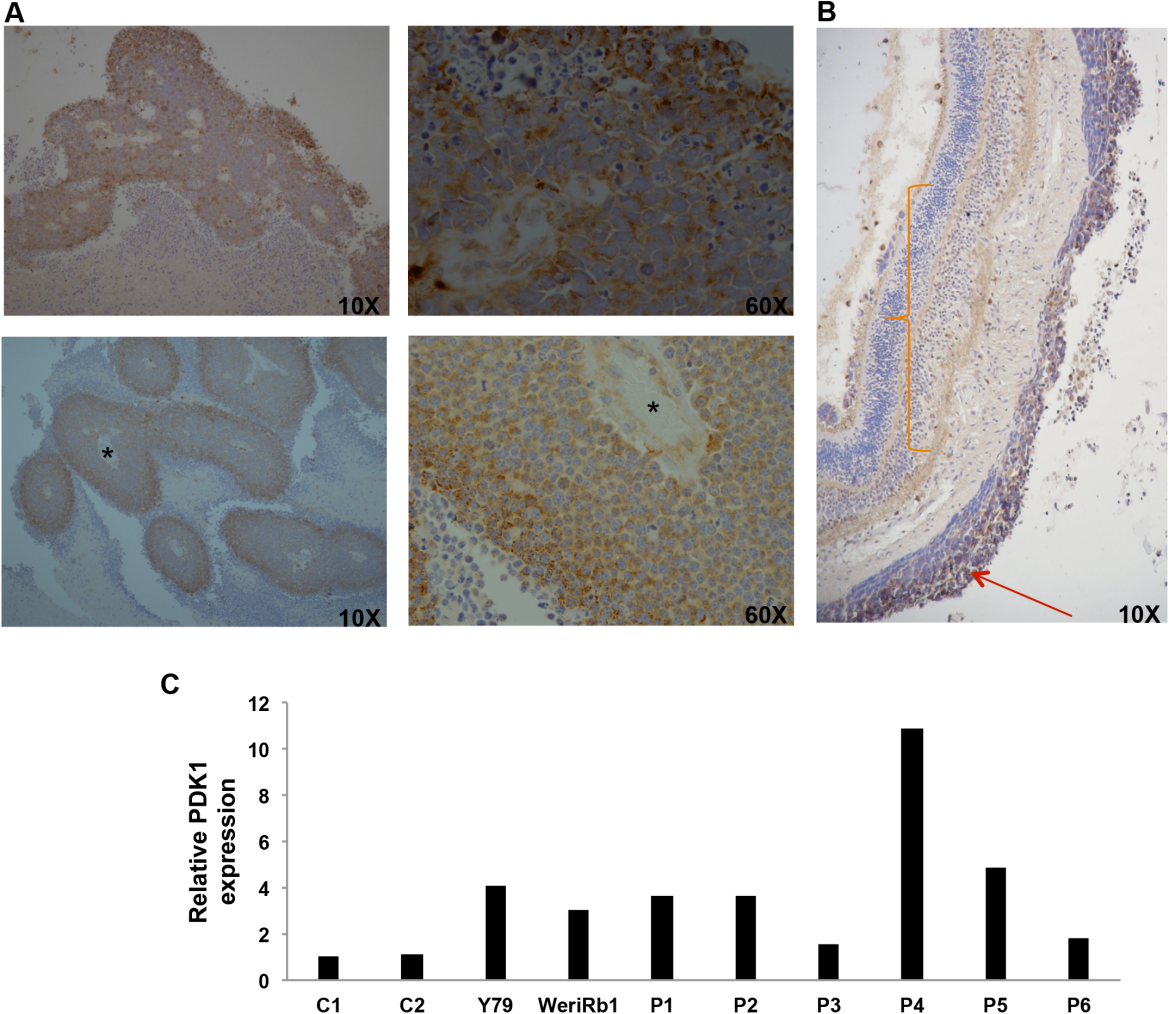
Expression of PDK1 in retinoblastoma tissues. (A) Immunohistochemistry (IHC) showing expression of PDK1 in retinoblastoma specimens. * Indicates vessel lumen. (B) IHC showing poor expression of PDK1 in uninvolved retina and strong expression in RB tumor tissue. Orange curly bracket indicates the uninvolved retina and red arrow denotes the expression of PDK1 in RB tumor region. (C) Relative mRNA expression of PDK1 was compared between retinoblastoma tissues and control retina. C1-C2, control retina; Y79 and Weri-Rb1, retinoblastoma cell lines; P1-P6, retinoblastoma tumor tissues.

To understand the role of PDK1 in RB, we first evaluated the expression of PDK1 in human RB patient samples by IHC (n = 26). Immunohistochemical staining revealed the granular expression of PDK1 in the cytosol as well as focally along the nuclear membrane (Fig 1A). Among the 26 samples, 16 of them were found to be positive for PDK1 expression (61.5%). There was significantly stronger expression of PDK1 in the hypoxic zones of the peritheliomatous arrangement of the tumor cells and vitreous seeds (Fig 1A). The expression was stronger in the outer layers of cells, not only in intensity, but also in the size of granules. However, tumors in highly vascular regions such as tumor foci in the choroid exhibited no expression of PDK1. In addition, we compared the expression of PDK1 in the RB tumor and the adjacent normal appearing uninvolved retina and found significantly lesser expression of PDK1 in normal retina than RB tissue (Fig 1B).

To evaluate the expression of PDK1 in normal retinal tissues, mRNA expression of PDK1 was determined by real-time PCR using specific primers in fresh RB tumor samples (n = 6) and control retina (n = 2). In addition, expression of PDK1 was measured in RB cell lines Y79 and Weri-Rb1. PDK1 mRNA was significantly overexpressed in RB tumor samples as well as Y79 and Weri-Rb1 cells compared to normal retinal tissue (Fig 1C). The expression of PDK1 mRNA varied significantly in different tumor samples, ranging from 1.6 to 11 fold compared to control retina. Overall, this data suggests that PDK1 is induced in RB tumors compared to normal retina.

### Pharmacological inhibition of PDK1 reduced retinoblastoma cell growth

**Fig 2:**
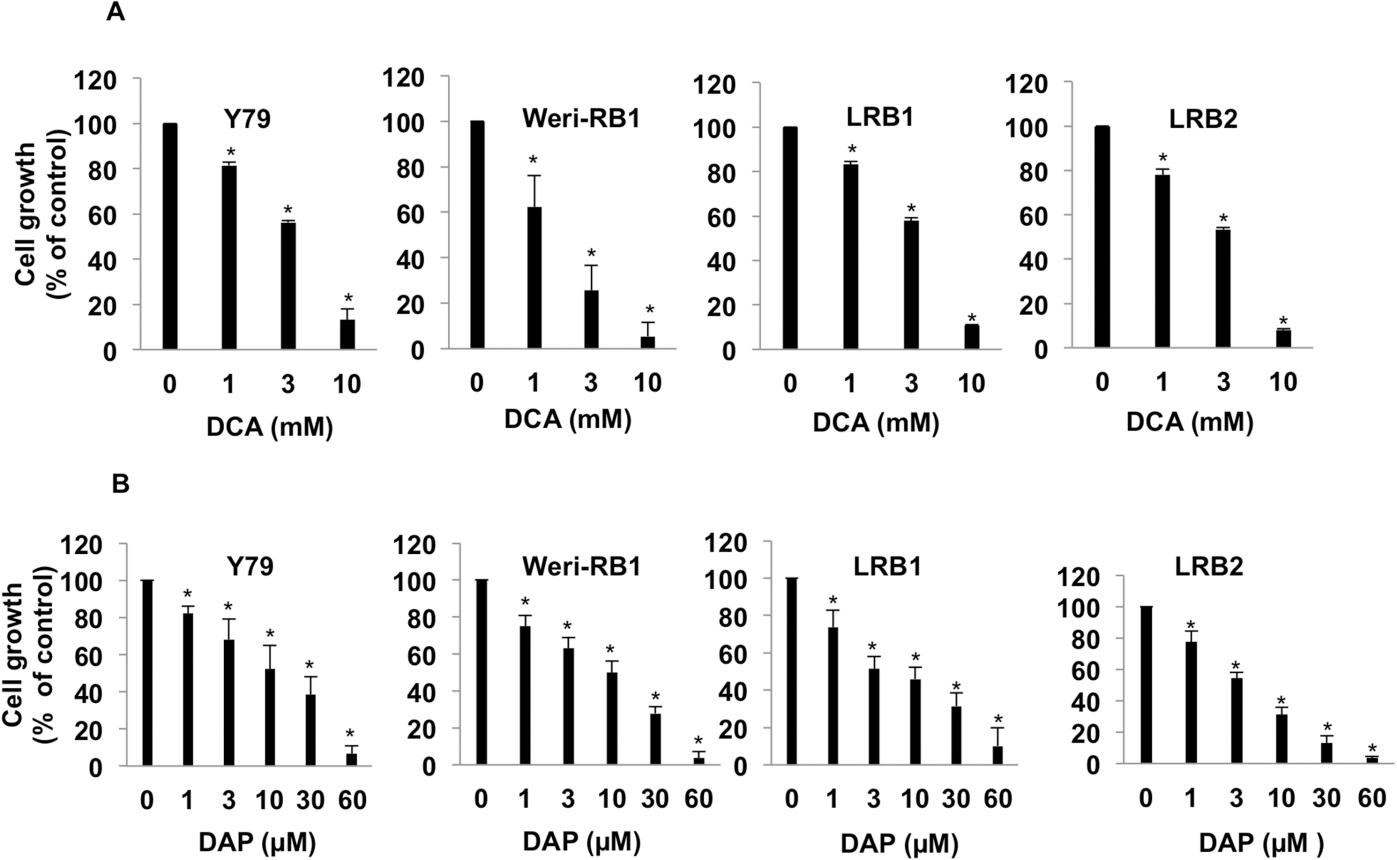
PDK1 inhibition results in decreased retinoblastoma cell growth. (A) Retinoblastoma cell lines (Y79 and Weri-Rb1) and patient derived cells (LRB1 and LRB2) were treated with different concentrations of DCA and cell growth was measured and compared to untreated cells. (B) RB cells were treated with increasing concentrations of DAP and cell growth was measured and compared to control cells. * Indicates significant difference (p<0.05) between control and treatment.

Since, PDK1 was found to be overexpressed in RB; we tested the inhibition of PDK1 as a therapeutic strategy using small molecule inhibitor of pyruvate dehydrogenase kinase, dichloroacetic acid (DCA). There are only two cell lines available commercially for RB with ATCC (Y79 and Weri-Rb1). Both these cell lines were used to study the inhibition of cell growth upon DCA treatment. In addition, primary human RB cells (LRB1 and LRB2) were treated with increasing concentrations of DCA and cell growth was measured. It was found that DCA effectively reduced cell growth, which correlated further with increasing concentrations of DCA. We observed that at 3 mM DCA, the cell growth compared to untreated cells was 56, 25, 58 and 53% in Y79, Weri-Rb1 cells and RB tumor derived cells LRB1 and LRB2, respectively. The cell growth was almost negligible at 10 mM and was found to be 13, 5, 11 and 8% correspondingly in Y79, Weri-Rb1, LRB1 and LRB2 (Fig 2A).

Moreover, Dichloroacetophenone (DAP), a highly potent inhibitor of PDK1 has been showed earlier to have an inhibitory effect in acute myeloid leukemia [15]. We studied the effect of DAP on RB cell growth and found that DAP could inhibit RB growth even at micromolar concentrations (Fig 2B). Further to study the inhibitory action of DCA on untransformed cells, we treated peripheral blood mononuclear cells (PBMCs) and retinal pigment epithelial cells (ARPE19) with same concentrations that were used to inhibit RB cell growth and found that DCA does not significantly change the viability of PBMCs or ARPE19 cells (S1 Fig).

### Combination effect of DCA and carboplatin on retinoblastoma cell growth

**Fig 3:**
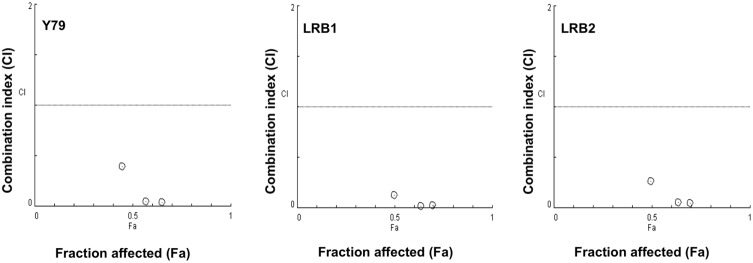
Combination activity of DCA and carboplatin on retinoblastoma cell growth. Y79 and patient derived retinoblastoma cells (LRB1 and LRB2) were treated with DCA or carboplatin alone or combination of DCA and carboplatin at various concentrations. Cell growth was measured by MTS assay and combination indices (CI) were calculated. CI = 1 –additive effect; CI<1 –synergistic effect and CI>1 –antagonistic effect.

Next, we determined if DCA could enhance the therapeutic potential of chemotherapy agents used in the treatment of RB. Retinoblastoma cell line Y79 and patient derived cells (LRB1 and LRB2) were treated with DCA or carboplatin alone or combination of DCA and carboplatin to study the efficacy of combination treatment on RB cell growth. The IC_50_ concentration for each drug was calculated individually and found to be in the range of 3.5–5.0 mM and 250–350 μM for DCA and Carboplatin respectively. Combination studies were performed at concentrations lower than IC_50_ of drugs. DCA when used in combination with carboplatin showed a combination index of less than one in Y79 cells suggesting a synergistic inhibition on RB cell growth. The decrease in cell growth when treated with DCA or carboplatin alone was less significant compared to combination treatment. Further, RB primary cells showed a similar synergistic effect upon combination treatment (Fig 3). These data indicate that DCA could enhance the therapeutic efficacy of carboplatin in RB.

### PDK1 inhibition led to increased apoptosis and decreased cell migration

**Fig 4:**
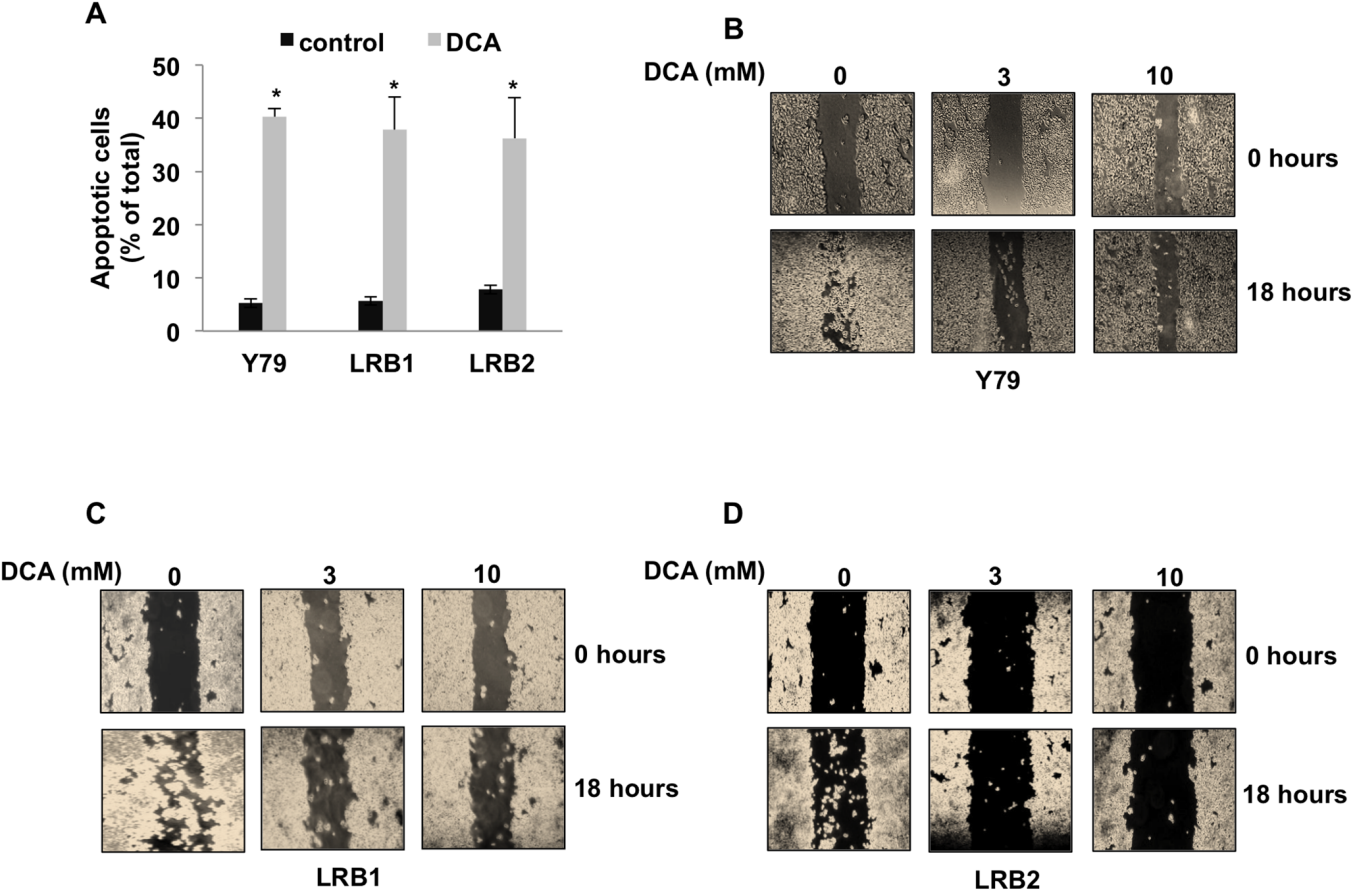
Inhibition of PDK1 induces apoptosis and decreases cell motility. Y79 and patient derived retinoblastoma cells (LRB1 and LRB2) were treated with DCA and (A) apoptosis and (B, C and D) cell migration were measured. * Indicates significant changes (p<0.05) between control and treatment.

Retinoblastoma cells (Y79, LRB1 and LRB2) were treated with 10 mM DCA for 48 hours and apoptosis was measured by using flow cytometry and annexin V and PI staining. PDK1 inhibition resulted in a significant increase in apoptosis. We observed that in Y79 cells, DCA treatment led to 8-fold increase in apoptosis and in patient samples (LRB1 and LRB2) similar results were observed with 7- and 9-fold increase respectively (Fig 4A).

In addition, the migration ability of Y79 cells and retinoblastoma patient derived cells was studied by migration assay in response to PDK1 inhibition. Cells were layered on poly-L-lysine coated slides; fine scratches were made and treated with different concentrations of DCA. The healing of scratch was photographed over a period of time. At 18 hours, in untreated Y79 cells the scratch was completely repopulated with cells. Whereas in DCA treated cells, there was very little to no migration (Fig 4B). In patient derived tumor cells (LRB1 and LRB2), similar results were observed (Fig 4C and 4D). However, there was a variation in the degree of migration. Overall, this data suggests that DCA treatment could inhibit migration of RB tumor cells.

### The effect of PDK1 inhibition on metabolic parameters

**Fig 5:**
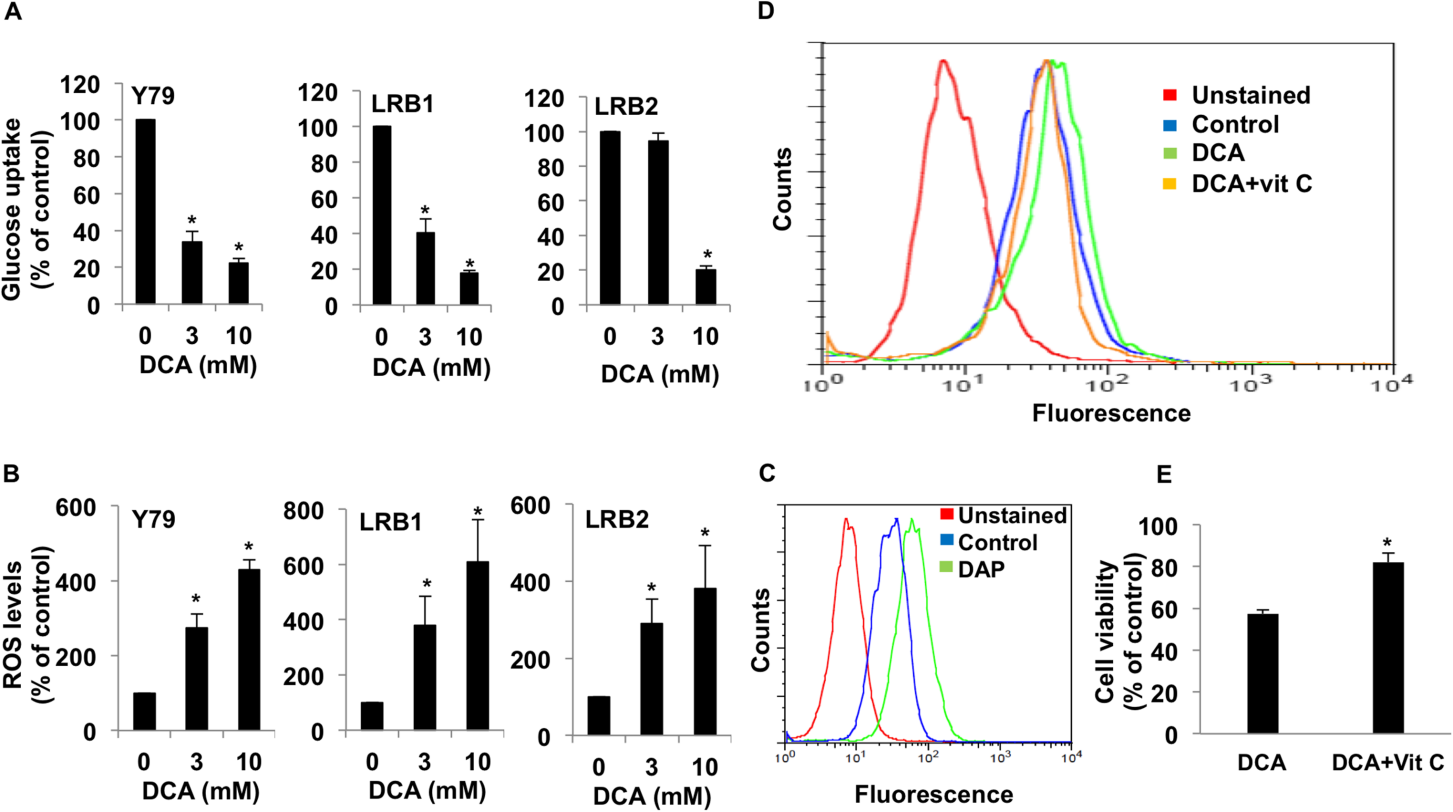
PDK1 inhibition alters metabolic parameters. Y79 and retinoblastoma primary cells (LRB1and LRB2) were treated with DCA and different metabolic parameters were measured. (A) Glucose uptake was measured using 2-NBD glucose. (B) Reactive oxygen species (ROS) levels were measured using DCF-DA. (C) ROS levels were measured by flow cytometry in response to inhibition with DAP. (D) RB cells were treated with DCA and DCA+vitamin C and ROS levels were measured and compared to control cells. (E) RB cells were treated with DCA and DCA+vitamin C and cell viability was measured and compared to control cells. * Indicates significant difference (p<0.05) between control and treatment.

In order to evaluate the effect of DCA treatment on tumor metabolism in RB, we measured glucose uptake, lactate and intracellular ROS levels. Glucose uptake was measured using 2-NBD glucose after 48 hours of incubation with DCA. The results were normalized to cell viability. Y79 cells showed significant reduction (33.62% of control at 3 mM and 22.33% of control at 10 mM) in glucose uptake at both concentrations tested. Even, RB tumor derived cells (LRB1 and LRB2) showed significant decrease in glucose uptake upon treatment with DCA (Fig 5A).

Next, we determined lactate levels after treatment of cells for 48 hours with DCA. Upon treatment with DCA there was a significant decrease in lactate levels compared to control cells (Table 1). These data implicate that RB cells have an elevated glycolysis and inhibition of PDK1 reversed the elevated glycolytic metabolism.

**Table 1:** Effect of DCA on lactate production in RB cells.

	Lactate (ng/μL)		
	Control	DCA (3 mM)	DCA (10 mM)
Y79	5.05	2.96	2.28
LRB1	5.37	3.11	2.55
LRB2	7.50	3.38	1.95

Treatment of RB cells with DCA leads to decreased lactate production. RB cells were treated with DCA for 48 hours and lactate levels were estimated. Upon DCA treatment, lactate levels were found to be reduced compared to control cells.

Further, we estimated ROS levels in tumor cells after 48 hours of PDK1 inhibition. DCA treatment led to an increased ROS (3- to 6-fold increase) (Fig 5B). In additional experiments we measured ROS levels in response to treatment with DAP and found that DAP significantly enhanced ROS levels (Fig 5C). These data provide additional evidence that inhibition of PDK led to decrease in glycolysis and an increase in mitochondrial metabolism of glucose through oxidative phosphorylation. Further to show that the observed changes in ROS levels were due to treatment with DCA, we treated cells with an anti-oxidant vitamin C and measured ROS and cell growth. It was observed that upon treatment with vitamin C, there was a reduction in ROS levels in DCA treated cells (Fig 5D). In addition a partial rescue in cell death was observed in the presence of vitamin C in DCA treated cells (Fig 5E).

### DCA treatment reduced PDK1 levels under hypoxia and inhibited Akt pathway

**Fig 6:**
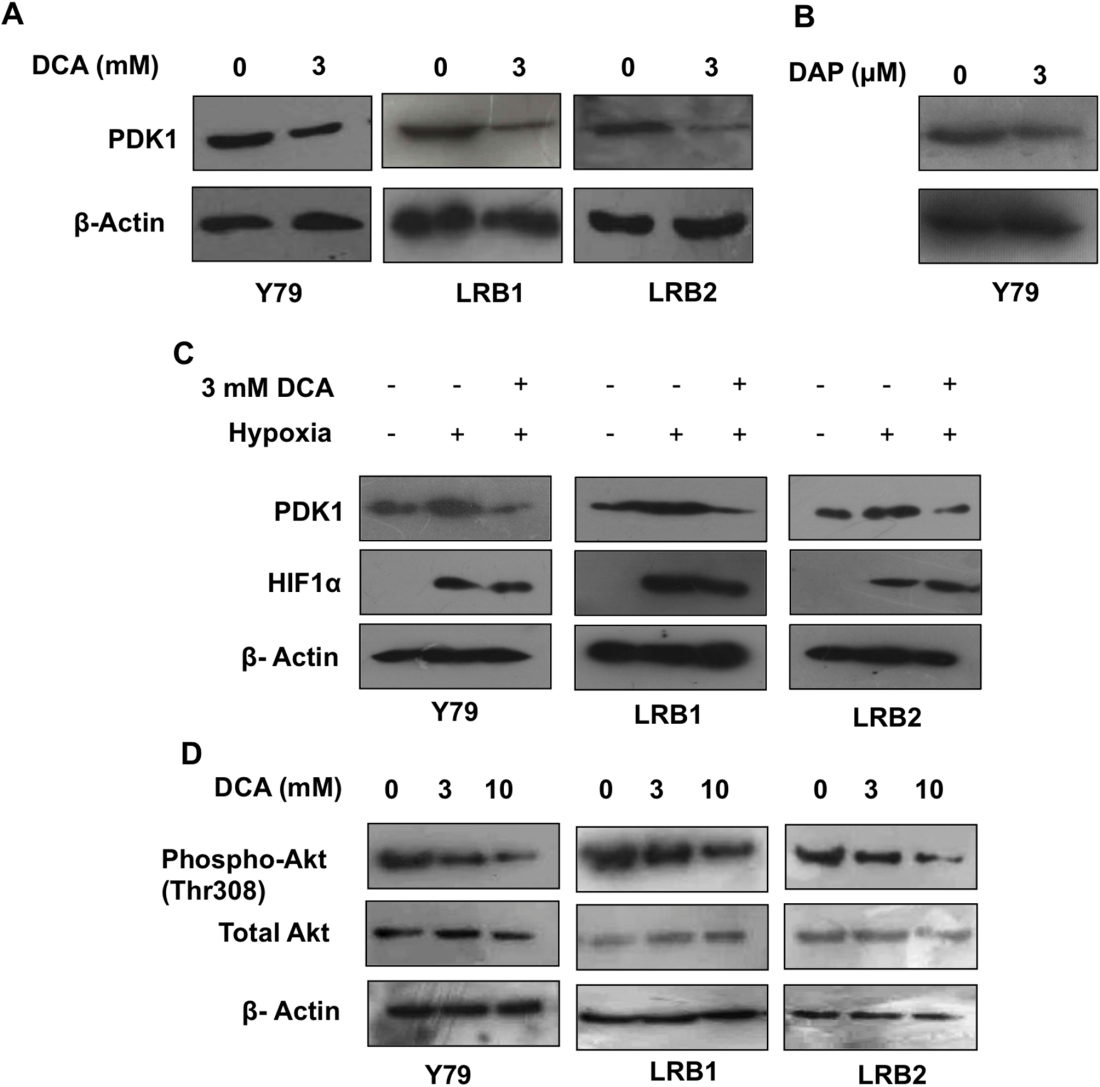
Effect of DCA treatment on PDK1 protein levels and PI3K/Akt pathway in RB. (A) DCA treatment decreases protein levels of PDK1. (B) Treatment of RB cells with DAP results in reduced levels of PDK1 protein. (C) Treatment of RB cells with DCA leads to decreased PDK1 protein levels under hypoxia. (D) DCA alters the activity of PI3K/Akt pathway in RB cells.

To study the mechanism of action of DCA on RB cell growth, we determined the expression of PDK1 protein levels upon DCA and DAP treatment. When cells were treated with 3 mM DCA for 48 hours, the protein expression of PDK1 was significantly reduced (Fig 6A) indicating that DCA could possibly be acting through inhibition of PDK1. Similar reduction in PDK1 protein levels was observed with DAP treatment (Fig 6B). We also found stronger expression of PDK1 in hypoxic regions and vitreous seeds, therefore in additional experiments; we tested the efficacy of PDK1 inhibition under hypoxic conditions. Cells (Y79, LRB1 and LRB2) were maintained under hypoxia (0.5% O_2_ level) or treated with hypoxic mimetic cobalt chloride and studied the expression of HIF1α, and PDK1. The expression of PDK1 and HIF1α was induced in the presence of hypoxia and cobalt chloride; however, the expression of PDK1 has decreased when cells were treated with DCA, but no change was observed in HIF1α levels (Fig 6C and S2 Fig). These data indicate that DCA treatment can alleviate changes associated with tumor-mediated hypoxia. Further, our data elucidated the changes in Akt signaling pathway after DCA treatment. Immunoblot assay revealed that DCA treatment led to reduced phosphoprotein levels of Akt (Thr308), whereas the total Akt levels remained unchanged (Fig 6D). Overall, these data indicate that DCA can act by either reducing PDK1 levels and/or inhibiting the Akt pathway.

### Consensus HIF1, Hypoxia Responsive element, E2F and MYCN binding elements are present in PDK1 gene sequence

We analyzed approximately 2 kb of DNA sequence upstream and downstream of transcription start site of PDK1 for consensus transcription factor binding sites. Precisely, human genomic contig NC_000002.12; and sequence from 172,553,960 to 172,557,892 was used for analysis. Consensus binding sites for E2F transcription factors, HIF1α, hypoxia responsive element (HRE) and MYCN were found in the sequence analyzed (S3 Fig), suggesting, PDK1 expression is coupled to RB-E2F pathway axis.

## Discussion

The present study for the first time demonstrated an elevated expression of PDK1 protein levels in RB tumors especially in vitreous seeds and hypoxic regions, signifying that PDK1 could be a potential therapeutic target in RB. The RB tumors with vitreous seeds and hypoxic regions are considered difficult to treat [6]. Identifying pathways that get activated under hypoxia is crucial for developing new drug targets. Our study showed that PDK1 inhibition could be a potential therapeutic target in RB even under hypoxic conditions. Our sequence analysis shows that PDK1 expression, possibly, is linked to RB-E2F pathway axis, and targeting PDK1 might result in sustained therapeutic response. In few earlier studies, the role of E2F transcription factors in the regulation of metabolic enzymes has been documented [16–18]. Besides, DCA treatment resulted in significant decrease in cell motility, which further argues in favor of targeting PDK1 in RB. Earlier studies using RB tumor specimens showed an elevated expression of certain genes involved in metabolic reprogramming [19–21]. These studies hint at targeting genes involved in altered metabolism for the treatment of RB. Consequently, inhibition of tumor cell growth using glycolysis inhibitor 2-deoxyglucose to control RB tumor growth was successfully attempted in mouse models [22,23]. The inhibition of PDK1 by DCA was achieved at millimolar concentration and is similar to other studies [24]. Further, DCA treatment on PBMCs or retinal pigment epithelial cells did not show any significant loss of cell viability indicating that DCA inhibition has no considerable effect on normal control cells, and a therapeutic window to treat cancer cells specifically has been demonstrated. In additional experiments we used a recently reported potent inhibitor of PDK1, dichloroacetophenone [15] and showed that PDK1 inhibition could be achieved even at micromolar concentrations in RB and this is especially interesting as it is always desirable to have an inhibitor that works at a lower concentration. Therapeutic efficacy of PDK1 inhibition by DCA has been showed for other tumors [25,26] including glioma [27], melanoma [28]. Further, the role of PDK1 and related isoforms in drug resistance and disease prognosis has been documented—Elevated expression of PDK1 was shown to be linked with malignant phenotype and poor prognosis in head and neck squamous cell carcinoma [24,29]. Upregulation of PDK1 and PDK3 led to therapy related drug resistance [30]. Overexpression of PDK3 and PDK4 was shown to be associated with drug resistance and early recurrence in colon cancer cells [31,32]. Interestingly, it was noted that, glioblastoma patients treated with DCA as an oral drug for 15 months in a clinical trial showed tumor regression, radiological stabilization and good safety profile [33]. Also a phase 1 trial to study DCA as a treatment in advanced solid tumors showed a decrease in (18) F-FDG uptake with length of DCA therapy [34]. Based on our analysis of the studies cited above and our results presented here strongly subscribe to the idea that PDK1 can be a target for controlling RB cell growth.

Chemotherapy has been successful in controlling retinoblastoma in a majority of children. However, it has been shown to have undesired side effects and also not all children respond to chemotherapy. Nevertheless, currently chemotherapy cannot be replaced completely with new drugs. But, combination of novel molecules with the existing chemotherapy may provide new treatment options with increased cure rates. Therefore, we tested the combination efficacy of DCA and carboplatin, a chemotherapy agent for RB in controlling RB cell growth. Our results show that DCA enhances the chemotherapeutic efficacy of carboplatin. Previous studies using combination of DCA and taxol were observed to have a synergistic effect on taxol-resistant oral cancer cells [35]. Additional studies have established that DCA enhanced the therapeutic efficacy of platinum compounds [36]. In addition, our study showed that inhibition of PDK1 led to increased apoptosis, decreased migration and changes in various metabolic parameters, signifying that inhibition of PDK1 could reverse certain changes associated with retinoblastoma tumor progression.

The inhibition of cell growth by DCA could possibly be because of several mechanisms. Our data suggest that DCA achieved this desired inhibitory effect by two possible mechanisms, 1. by causing decrease in PDK1 protein levels, 2. by inhibition of PI3K/Akt pathway. Most notably, DCA also reduced PDK1 protein levels in the presence of hypoxia. This is especially interesting given the induced expression of PDK1 in hypoxic regions and vitreous seeds. However, the mechanism of action of DCA is still being investigated. DCA has been showed to have effects on several proteins including HIF1, survivin [37] and BCL2 family of proteins [38]. Further, DCA has been demonstrated to inhibit phosphorylation of Akt in colon cancer cells [39]. In addition, PDK1 was recognized to stabilize PI3K/Akt pathway [15]. On the other hand, Akt is also known to regulate MYCN—an important proto-oncogene in RB [40,41] and glycolysis by several mechanisms [42,43]. Though several studies exist on mechanism of DCA, it is still not clear how DCA can exert a plethora of effects. Based on literature and our study, we propose that inhibition of PDK1 by DCA is through an effect of the drug on levels and activity of PDK1, and PI3K/Akt/MYCN axis. This is supported by our finding that PDK1 promoter has consensus MYCN motifs and HIF1 biding sites. In conclusion, our results suggest PDK1 is overexpressed in RB and tumor growth could be attenuated by DCA treatment via inhibition of PI3K/Akt pathway and decrease in PDK1 protein levels. However, DCA cannot replace the existing chemotherapy, and we suggest that combination of DCA with chemotherapy may be a potential therapeutic option in RB.

## Supporting information

S1 Fig Effect of DCA treatment on cell viability of (A) Peripheral blood mononuclear cells (PBMCs) and (B) ARPE-19 cells.
(TIF)

S2 Fig Effect of DCA and Cobalt Chloride treatment on PDK1 protein levels. (TIF)

S3 Fig Promoter analysis of PDK1. DNA sequence (2 kb) upstream and downstream of transcription start site of PDK1 was used for analysis. The genomic contig NC_000002.12 and sequence from 172,553,960 to 172,557,892 were used. +1 indicates transcription start site. Red box–E2F binding site; Green box–HIF1 binding site; Yellow box: HRE binding site; Purple box: MYCN motifs.
(TIF)
