# Supplementary figures and images for: Overexpression of pyruvate dehydrogenase kinase 1 in retinoblastoma: A potential therapeutic opportunity for targeting vitreous seeds and hypoxic regions
Source: PLoS One. 2017 May 15;12(5):e0177744. doi: 10.1371/journal.pone.0177744 (PMC5432179; doi:10.1371/journal.pone.0177744)

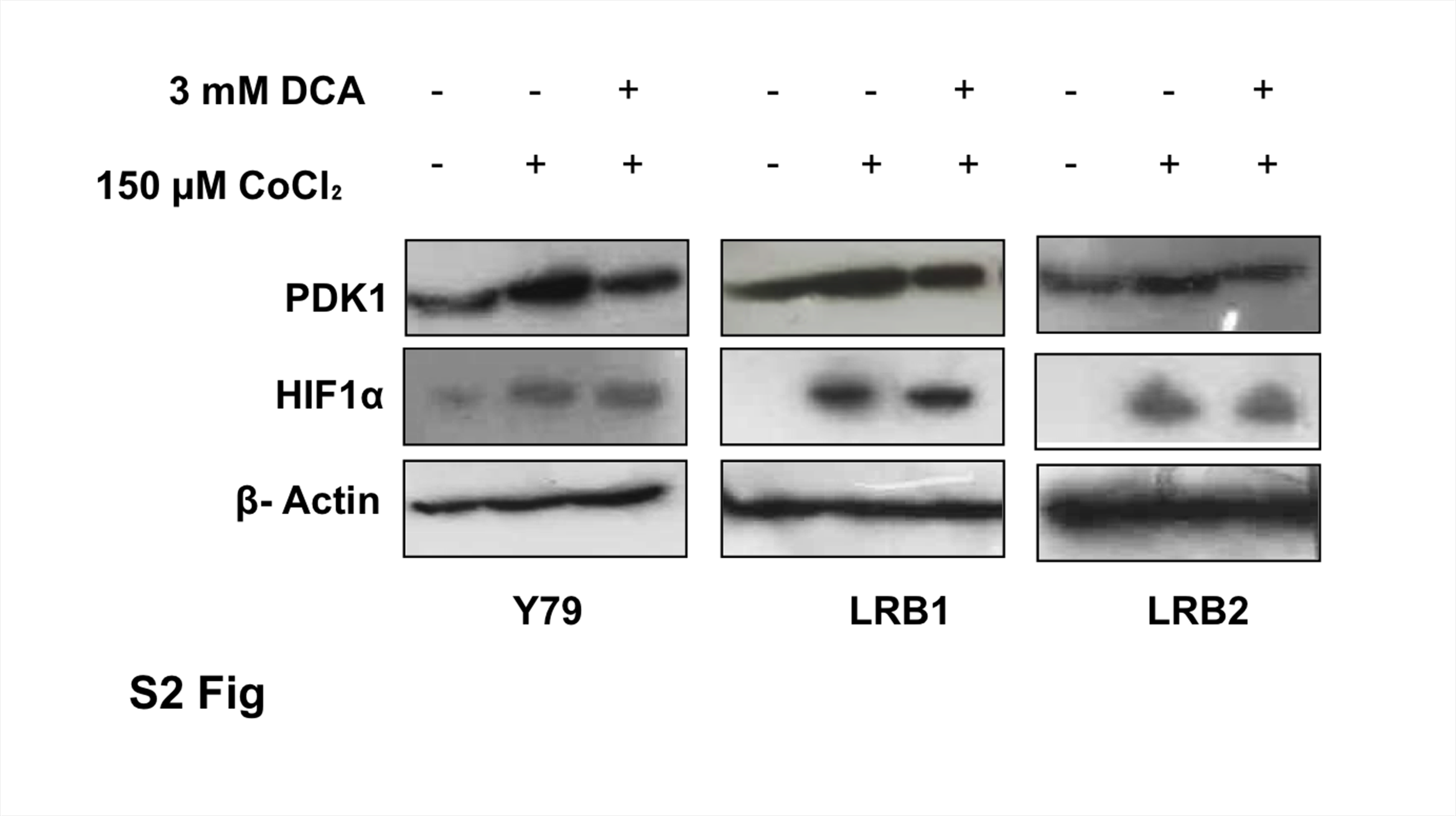

Supplement: S2 Fig — (TIF) [file pone.0177744.s002.tif]

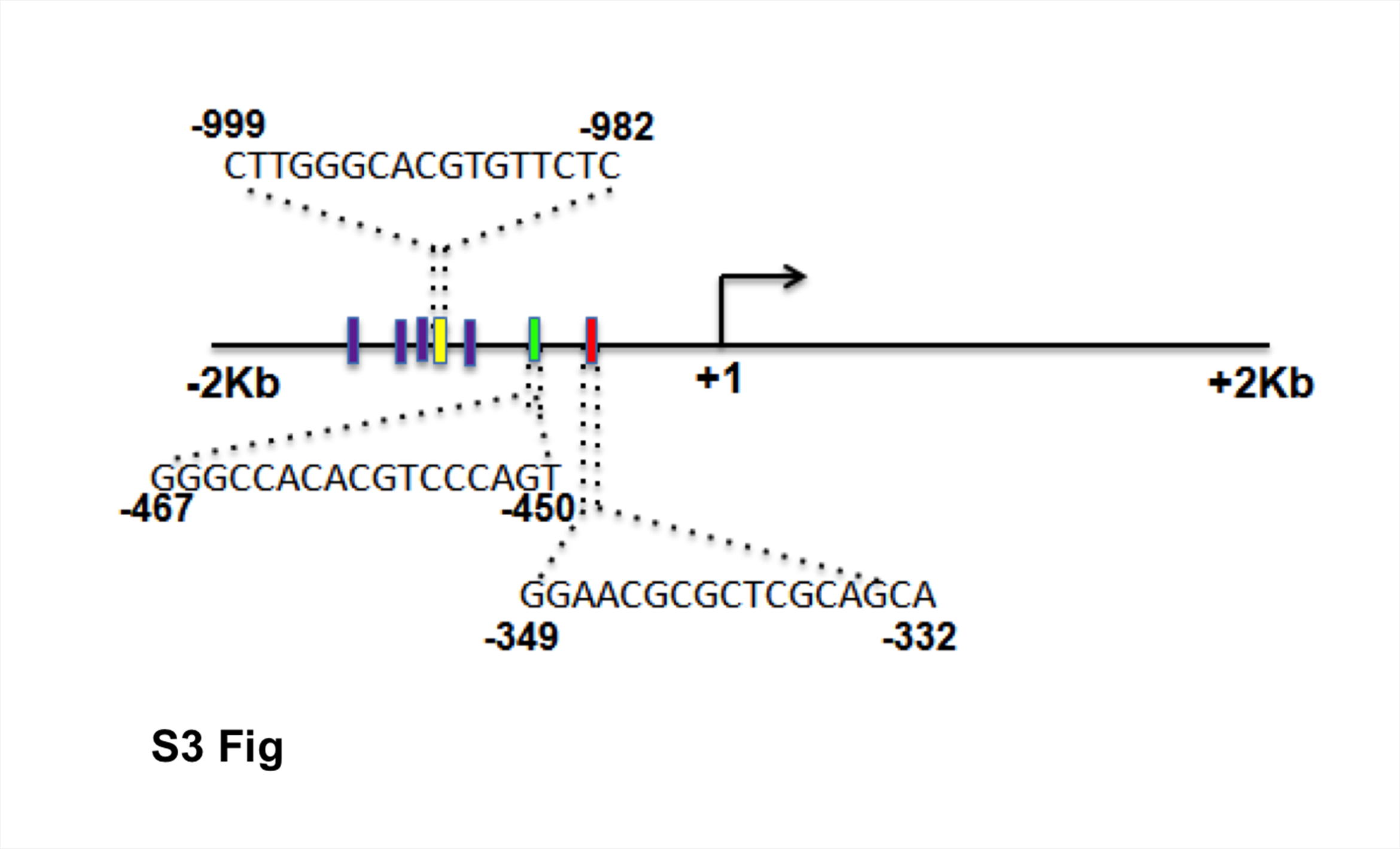

Supplement: S3 Fig — DNA sequence (2 kb) upstream and downstream of transcription start site of PDK1 was used for analysis. The genomic contig NC_000002.12 and sequence from 172,553,960 to 172,557,892 were used. +1 indicates transcription start site. Red box–E2F binding site; Green box–HIF1 binding site; Yellow box: HRE binding site; Purple box: MYCN motifs. (TIF) [file pone.0177744.s003.tif]
